# Pseudoceratonic Acid and Moloka’iamine Derivatives from the Red Sea Verongiid Sponge *Pseudoceratina arabica*

**DOI:** 10.3390/md18110525

**Published:** 2020-10-23

**Authors:** Lamiaa A. Shaala, Diaa T. A. Youssef

**Affiliations:** 1 King Fahd Medical Research Center, Natural Products Unit, King Abdulaziz University, Jeddah 21589, Saudi Arabia; 2 Department of Medical Laboratory Sciences, Faculty of Applied Medical Sciences, King Abdulaziz University, Jeddah 21589, Saudi Arabia; 3 Suez Canal University Hospital, Suez Canal University, Ismailia 41522, Egypt; 4 Department of Natural Products, Faculty of Pharmacy, King Abdulaziz University, Jeddah 21589, Saudi Arabia; dyoussef@kau.edu.sa; 5 Department of Pharmacognosy, Faculty of Pharmacy, Suez Canal University, Ismailia 41522, Egypt

**Keywords:** Red Sea Verongiid sponge, *Pseudoceratina arabica*, pseudoceratonic acid, dibromotyrosine alkaloids, ceratinines N and O, structure determinations, antibiotics and cytotoxic activities

## Abstract

During an investigation of the chemistry of the Red Sea Verongiid sponge *Pseudoceratina arabica,* we discovered a small molecule, pseudoceratonic acid (**1**), along with the new moloka’iamine derivatives, ceratinines N (**2**), O (**3**), and the previously reported compounds moloka’iamine (**4**), hydroxymoloka’iamine (**5**) and ceratinamine (**6**). The structural assignments of **1**–**6** were accomplished by interpretation of their NMR and HRESIMS spectral data. Pseudoceratonic acid possesses a dibrominated hydrazine-derived functional group not found in any reported chemical compound. Pseudoceratonic acid selectively inhibited the growth of *E. coli* and *S. aureus*, while ceratinine N selectively inhibited *C. albicans*. Further, ceratinine N showed potent cytotoxic effects against the triple-negative breast cancer, colorectal carcinoma, and human cervical carcinoma cell lines down to 2.1 µM.

## 1. Introduction

As a result of the global and expanding resistance against today’s available antibiotics and antitumor therapies, novel chemical entities with antibiotic and anticancer potential are in high demand to support the clinical pipelines with enough drug leads. Infections with *Staphylococcus aureus*, malaria, and Tuberculosis represent a few examples of diseases that have become difficult to cure with current antibiotics [1]. Therefore, there is an urgent need for new drugs/drug leads to combat expanding drug resistance. Further, the declining number of FDA-approved candidates has warranted a renewed interest in the role of secondary metabolites with the goal of characterizing new and bioactive structural scaffolds. Marine organisms represent an unlimited foundation of secondary metabolites with antibiotic and antitumor potential.

Sponges of the Verongida order are well-known for producing bromotyrosine-derived secondary metabolites with diverse bioactivities [2,3,4,5,6]. Bromotyrosines include several classes of brominated marine alkaloids such as spirocyclohexadienylisoxazolines, spirooxepinisoxazolines (psammaplysins), oximes, bastadins, and many other compounds [5]. Members of these classes have been associated with diverse bioactivities including antiviral [7], antimicrobial [8,9,10], antifungal [11], anticancer [12,13,14,15], antimigratory [14], antimalarial [16,17,18], parasympatholytic [8], enzyme inhibition [19] and antifouling properties [20,21].

As a continuation of our interest in bioactive chemical entities from Red Sea Verongiid sponges [8,22], a new sample of the sponge *Pseudoceratina arabica* was collected and investigated. The current study aimed to identify the cytotoxic and antimicrobial compounds from the methanolic extract of the sponge. Fractionation and purification of the active fractions gave pseudoceratonic acid (**1**), ceratnines N (**2**) and O (**3**) along with moloka’iamine (**4**) [2], hydroxymoloka’iamine (**5**) [8] and ceratinamine (**6**) [6]. Herein, we report on the purification, structural determinations, and the biological effects of compounds **1**–**6**.

## 2. Results and Discussion

### 2.1. Purification of Compounds ***1**–**6***

Extraction of the freeze-dried sponge *P. arabica* with MeOH, followed by partition of the resulted extract between H_2_O-MeOH and n-hexanes, CH_2_Cl_2_ and EtOAc gave three organic fractions. The EtOAc-soluble fraction was subjected to chromatographic fractionation on reversed phase (C18) Sep-Pak cartridge and Sephadex LH-20. Purification of the active fractions on C18 HPLC afforded compounds **1**–**6**.

### 2.2. Structural Determination of ***1**–**6***

**Figure 1:**
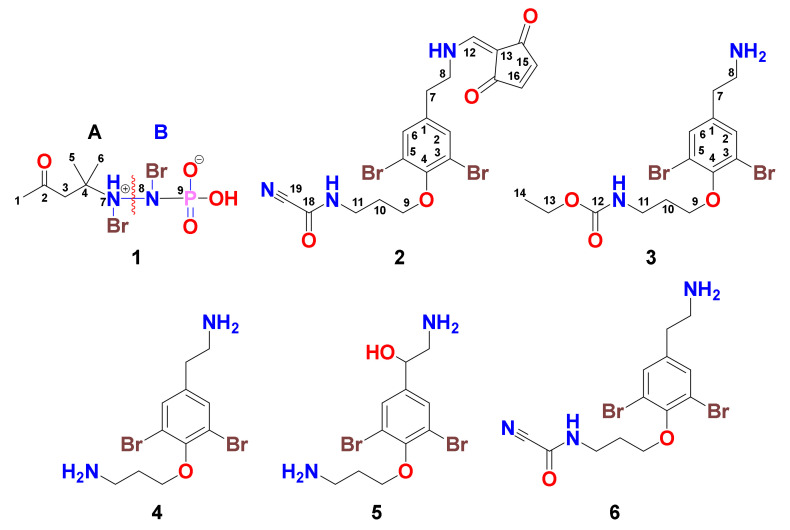
Structures of compounds **1**–**6**.

The positive ESIMS of **1** showed three ion peaks at *m/z* 366.9, 368.9 and 370.9 (1:2:1), supporting the existence of 2 bromine atoms in **1**. The molecular formula C_6_H_13_^79^Br_2_N_2_O_4_P was obtained from the ion peak at 366.9064 [M + H]^+^ in the positive HRESIMS (Figure S1). Also, the negative HRESIMS (Figure S2) of **1** confirmed the molecular formula. Compound **1** (Figure 1) was assigned by interpretation of its 1D (^1^H and ^13^C) (Figures S3 and S4) and 2D NMR spectral data including multiplicity-edited HSQC (Figure S5), and ^1^H-^13^C (Figure S6) and ^1^H-^15^N HMBC (Figure S7) experiments. These combined data, along with the HRESIMS and MS fragmentation of **1**, supported the structural determination of two subunits in **1** including 4-(bromoamino)-4-methylpentan-2-one (A) and *N*-bromo-phosphoramidic acid (B) connected together through *N*-7 and *N*-8 (Figure 1).

**Figure 2:**
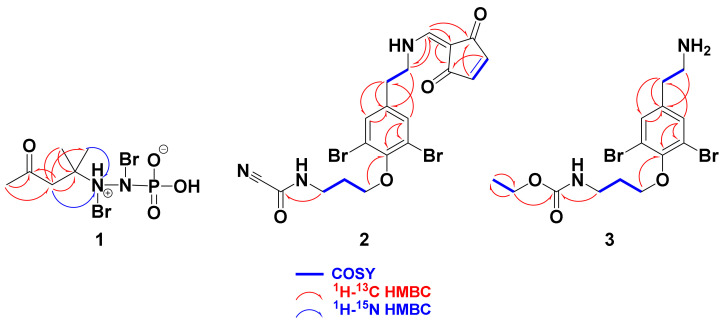
COSY, ^1^H-^13^C HMBC of **1**–**3** and ^1^H-^15^N HMBC of **1**.

**Figure 3:**
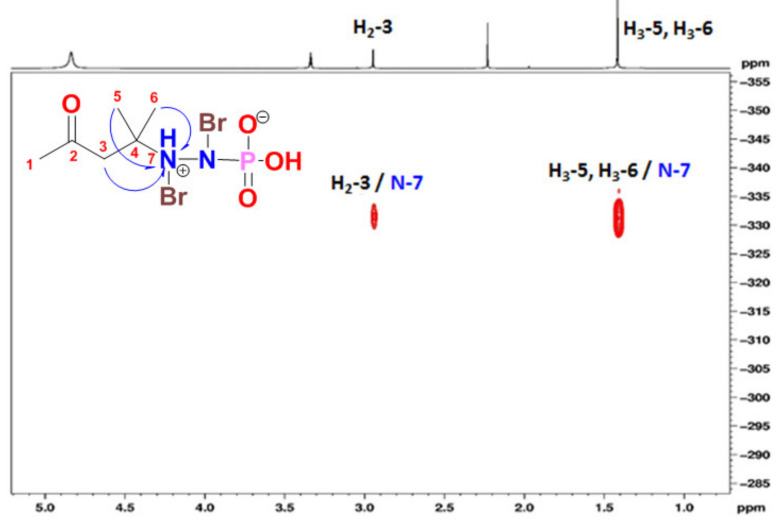
^1^H-^15^N HMBC spectrum of **1**.

**Figure 4:**
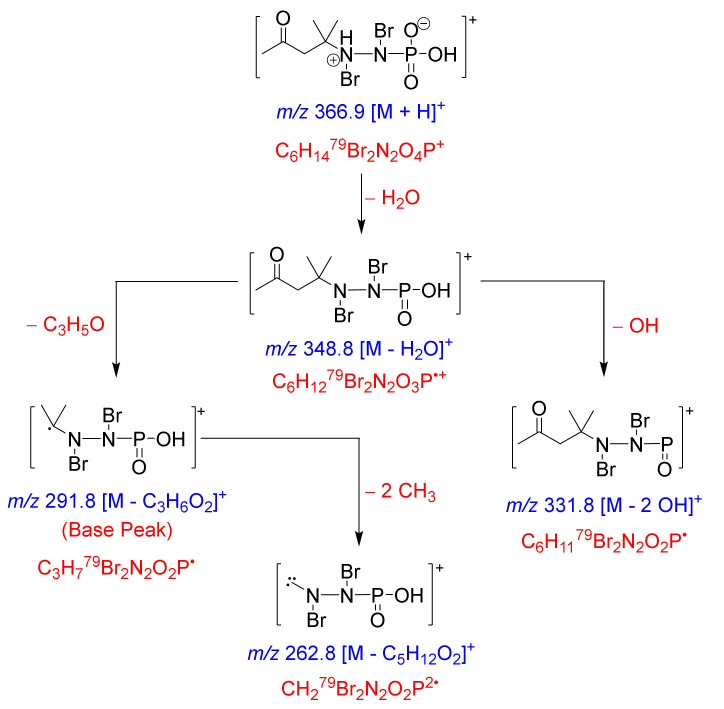
Important MS fragmentation ion peaks of **1**.

The ^1^H NMR spectrum of **1** showed three singlets in the ratio of 1.5:1.0:3.0 (3.0:2.0:6.0) at δ_H_ 2.23 (3H, s), 2.91 (2H, s) and 1.41 (6H, s) corresponding to H_3_-1, H_2_-3 and H_3_-5/6 (Table 1). In the HSQC, these signals are correlated to the ^13^C signals at δ_C_ 31.0 (CH_3_, C-1), 50.7 (CH_2_, C-3) and 26.0 (2 × CH_3_, C-5 and C-6), respectively. The quaternary signals at δ_C_ 209.3 and 53.4 (Table 1) were assigned to a ketone (C-2) and a quaternary carbon (C-4) attached to a nitrogen atom (*N*-7) as supported by HMBC correlations from H_2_-3 to C-2, from H_3_-1 to C-2 (δ_C_ 209.3), from H_2_-3 to C-4 (δ_C_ 53.4) and from H_3_-5/H_3_-6 to C-4. Furthermore, the ^1^H-^15^N HMBC cross-peaks from H_2_-3 to *N*-7 and from H_3_-5/H_3_-6 to *N*-7 supported the connection of *N*-7 to C-4 (Figure 2 and Figure 3). The fragmentation ion peaks of **1** (Figure 4) supported the *N*-bromination of part A. Thus, fragment A was assigned as 4-(bromoamino)-4-methylpentan-2-one.

The molecular formula of fragment A is counted for “C_6_H_11_BrNO”. Thus, the remaining elements of **1** “H_2_BrNO_3_P” are counted for an *N*-bromophosphoramidic acid moiety (Fragment B), as confirmed from the fragmentation ion peaks of **1** (Figure 4 and Figure S8). The fragment ion peaks at *m/z* 348.8, 350.8 and 352.8 (1:2:1) resulted from the loss of OH from the phosphoramidic acid [M + H − H_2_O]^+^ (Figure 4). Additional loss of C_3_H_5_O fragment from the other terminus of **1** gave a base peak at *m/z* 291.8/293.8/295.8 (1:2:1) [M + H − H_2_O − C_3_H_5_O]^+^. The loss of another OH from the ion peak at *m/z* 348.8 resulted in an ion peak at *m/z* 331.8/333.8/335.8 [M + H – H_2_O − OH]^+^. Finally, the loss of two methyls from the base peak at *m/z* 291.8/293.8/295.8 gave a minor ion peak at *m/z* 262.8/264.8/266.8 [M + H − C_5_H_12_O_2_]^+^ (Figure 4).

The existence of the fragmentation ion peaks at *m/z* 348.8, 331.8 and 291.8 and 262.8 supported the existence of both subunits (A and B), the dibromination of the nitrogen atoms in **1** and the linkage between *N*-7 and *N*-8. Accordingly, compound **1** was assigned as shown in Figure 1 and named pseudoceratonic acid.

**Figure 5:**
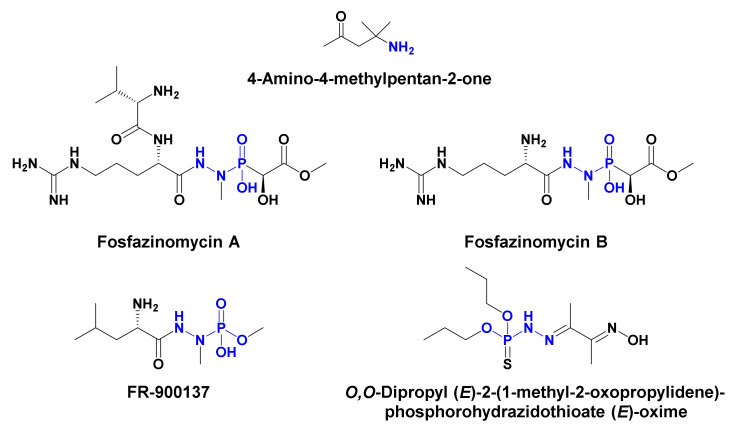
Microbial-derived compounds with similar subunits in **1** [27,28,29,30,31].

Hydrazine derivatives and other dinitrogen-containing features are commonly found in natural compounds of terrestrial, microbial, and marine origin [23,24]. Similarly, derivatives of phosphoramidic acid are represented in different chemical entities of natural origin [25,26]. A literature search revealed only four compounds with *N*-*N*-linked-phosphorous moiety including fosfazinomycins A and B from *Streptomyces lavendofoliae* [27], FR-900137 from *Streptomyces unzenensis* [28,29] and *O,O*-dipropyl (*E*)-2-(1-methyl-2-oxopropylidene)phosphorohydrazidothioate (*E*)-oxime) from the dinoflagellate *Gymnodinium breve* [30]. Similarly, 4-amino-4-methylpentan-2-one (part A in **1**) was previously reported from *Streptomyces pleomorphus* [31] (Figure 5). The existence of such microbial-derived compounds supports the hypothesis of the microbial origin of pseudoceratonic acid.

**Figure 6:**
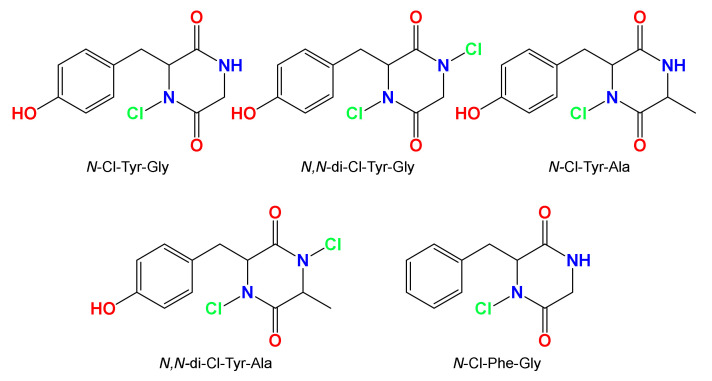
Chemical structures of *N*-chlorinated dipeptides [32].

Compound **1** has several features that are not found in previously reported natural products. Perhaps most spectacularly, the dibromination of the hydrazine part, a feature that has only been reported in a few synthetic compounds that were not NMR-characterized. *N*-Halogenated compounds are extremely rare. Recently, several *N*-chlorinated dipeptides (Figure 6), which again were not NMR-characterized, were discovered as disinfection byproducts resulted from chlorination of drinking water [32]. Finally, since compound **1** shares most of its functionalities with other microbial-derived compounds [27,28,29,30,31], we speculate that **1** could be a microbial product that is released as a part of a defense mechanism of the sponge after its cutting underwater.

The existence of three pseudomolecular ion peaks at 509.9, 511.9, and 513.9 (1:2:1) [M + H]^+^ in the ESIMS of **2** confirmed two bromine atoms in **2** (Figure 1). Its molecular formula C_19_H_17_^79^Br_2_N_3_O_4_ was confirmed from the ion peak at *m/z* 509.9669 [M + H]^+^ in the positive HRESIMS (Figure S9), confirming 12 degrees of unsaturation. Interpretation of its ^1^H (Figure S10) and ^13^C NMR (Figure S11) spectra along with the DEPT (Figure S12), COSY (Figure S13), HSQC (Figure S14) and HMBC (Figure S15) confirmed the assignment of three substructural units (A–C). The ^1^H and ^13^C NMR signal at δ_H/C_ 138.6 (qC, C-1), 7.54 (2H, s)/134.8 (2 × CH, C-2,6), 119.3 (2 × CH, C-3,5), 153.2 (qC, C-4), 2.86 (2H, t)/36.7 (CH_2_, C-7), 3.61 (2H, t)/51.9 (CH_2_, C-8), 4.02 (2H, t)/71.0 (CH_2_, C-9), 2.08 (2H, quin.)/30.1 (CH_2_, C-10) and 3.56 (2H, t)/38.4 (CH_2_, C-11) confirmed the presence of the moloka’iamine moiety (subunit A) [2] (Table 2). In addition, the resonances at δ_H/__C_ 7.21 (1H, H-12)/150.8 (CH, C-12), 99.5 (qC, C-13), 198.7 (qC, C-14), 6.67 (1H, H-15)/142.7 (CH, C-15), 6.73 (1H, H-16)/142.9 (CH, C-16) and 196.8 (qC, C-17) confirmed the presence of 2-(methylene)cyclopent-4-ene-1,3-dione part (subunit B) [7,14]. Finally, the signals at δ_C_ 145.0 (qC, C-18) and 113.1 (qC, C-19) confirmed the existence of a cyanoformamide moiety (subunit C) [6]. The linkage of subunits B and C to the terminal amines of the moloka’iamine at C-8 and C-11, respectively, was confirmed from HMBC of H_2_-8 (δ_H_ 3.61)/C-12 (δ_C_ 150.8) and from H-12 (δ_H_ 7.21)/C-8 (δ_C_ 51.8) as well as HMBC from H_2_-11 (δ_H_ 3.56) to C-18 (δ_C_ 145.0) (Figure 2 and Table 2), completing the molecular formula of **2** and its assignment. Accordingly, **2** was assigned as shown in Figure 1 and named ceratinine N.

The ESIMS spectrum of **3** with three ion peaks at 422.9, 424.9 and 426.9 (1:2:1) [M + H]^+^ supported 2 bromine atoms in the molecule. Compound **3** (Figure 1) showed molecular formula C_14_H_20_^79^Br_2_N_2_O_3_ as confirmed by the positive HRESIMS (422.9923, C_14_H_21_^79^Br_2_N_2_O_3_, [M + H]^+^) (Figure S16), supporting five degrees of unsaturation. Interpretation of the ^1^H (Figure S17), ^13^C (Figure S18), DEPT (Figure S19) spectra and the 2D (COSY, HSQC and HMBC) (Figures S20–S22) experiments of **3** allowed its assignment. The ^1^H and ^13^C spectra of **3** showed, beside the regular signals of the moloka’iamine moiety [2] (Table 2), additional signals at δ_H/C_ 159.2 (QC, C-12), 4.05 (2H, q, H_2_-13)/61.7 (CH_2_, C-13) and 1.22 (3H, t, H_3_-13)/14.5 (CH_3_, C-13). These signals were assigned for a terminal ethyl carbamate moiety [22,33]. The attachment of the carbamate moiety to the terminal amine at C-11 was confirmed by HMBC cross-peaks from H_2_-11 (δ_H_ 3.42) to C-12 (δ_C_ 159.2), H_2_-13 (δ_H_ 4.05) to C-12 (δ_C_ 159.2) and from H_3_-14 (δ_H_ 1.22) to C-13 (δ_C_ 61.7) (Figure 2 and Table 2), completing the structural assignment of **2**. Thus, compound **2** was assigned as shown in Figure 1 and named ceratinine O.

The antimicrobial evaluation of the compounds against *E. coli, S. aureus* and *C. albicans* at 50 µg/disc was carried out using disc diffusion assay. Pseudoceratonic acid (**1**) was the most active against *E. coli* and *S. aureus* with inhibition zones of 15 and 17 mm, respectively, while it displayed weak effect towards *C. albicans* with inhibition zone of 6 mm, suggesting selective antibacterial activity. On the other hand, ceratinine N (**2**) was more active against *C. albicans* (inhibition zone = 16 mm) and less active against the other pathogens, suggesting selectivity against *C. albicans*. The other compounds were weakly active against the three pathogens with inhibition zones of 6–9 mm (Table 3).

The minimal inhibitory concentrations (MICs) of the compounds were evaluated using a microdilution method (Table 3). Pseudoceratonic acid exhibited the highest activity against *E. coli* and *S. aureus* with MIC values of 16 and 16 µg/mL, respectively. Ceratinine N, on the other hand, displayed the highest antifungal activity against *C. albicans* with an MIC value of 16 µg/mL. 

The cytotoxic and antiproliferative effects of the compounds against MDA-MB-231, HeLa, and HCT 116 cell lines were evaluated using MTT (3-(4,5-Dimethylthiazol-2-yl)-2,5-diphenyltetrazolium bromide) assay (Table 4). Ceratinine N (**2**) demonstrated potent activities against the cell lines with IC_50_ of 3.1, 2.3 and 2.9 µM, respectively. Conversely, ceratinine O (**3**) displayed moderate activity towards HCT 116 with IC_50_ of 9.5 µM. The remaining compounds displayed IC_50_ values of ≥10 µM. Furthermore, **2** showed cytotoxicity towards NHDF (normal human dermal fibroblasts) cells with IC_50_ of 3.5 µM, suggesting marginal selectivity towards the tested cancer cell lines.

These results suggest that pseudoceratonic acid (**1**) possesses selectivity against *S. aureus* and *E. coli*, while ceratinine N (**2**) is selectively active against *C. albicans*. The potent cytotoxic and antiproliferative activities of ceratinine N (**2**) suggest the importance of 2-(methylene)cyclopent-4-ene-1,3-dione moiety for maximum cytotoxic activity.

## 3. Materials and Methods

### 3.1. General Experimental Procedures

HRESIMS spectra were acquired on Micromass Q-ToF spectrometer (Waters Corporation, Milford, MA, USA). NMR spectra were obtained on Bruker Avance DRX 400 and 600 MHz (Bruker, Rheinstetten, Germany). The UV spectra were measured on a Hitachi 300 spectrometer (Hitachi High-Technologies Corporation, Kyoto, Japan) and as reported earlier [34]. Liquid–Liquid partitions, C18 Sep-Pak and Sephadex LH-20 were used for fractionation of the extract and successive fractions. A Gemini^®^ C18 column (5 µm, Phenomenex, 250 × 0.64 mm) (Torrance, CA, USA) was used for HPLC purification of the compounds. The HCT 116, MDA-MB-231 cell lines were obtained from American Type Culture Collection (ATCC) (Manassas, VA, USA), while NHDF cell line was obtained from PromoCell GmbH (Heidelberg, Germany).

### 3.2. The Sponge Pseudoceratina arabica

*Pseudoceratina arabica* was collected via scuba diving (−13–17 m) from the Anas Reef (N 021°39′17.5″, E 038°52′26.3″) in the Saudi Red Sea. The sponge consists of an encrusting mass of 1–2 cm with a conulose surface and bright greenish-yellowish color underwater and greenish-yellow interior. A voucher specimen was stored at University of Amsterdam (code No. RMNHPOR 9161) and another specimen was kept at King Abdulaziz University (code No. KSA-58). A full description of the sponge is reported earlier [14].

### 3.3. Purification of ***1**–**6***

The lyophilized sponge materials (50 g) were macerated in MeOH overnight (3 × 500 mL). The dried methanolic extract was treated with 200 mL of 50% aqueous MeOH followed by partition against n-hexanes, CH_2_Cl_2_ and EtOAc. The cytotoxic EtOAc extract (IC_50_ = 5 µg/mL against HCT 116) was dried and the residue was subjected to partition on C18 cartridge (Sep-Pak, Waters, 1 g) using H_2_O-CH_3_CN-MeOH gradients. The fraction eluted with H_2_O-MeCN (8:2) (175 mg) was partitioned on Sephadex LH-20 affording five fractions (F1–F5). The antimicrobial fraction (F3, 24 mg) (inhibition zone = 9 mm against *E. coli*) was purified on C18 HPLC column (Gemini^®^ 5 µm, Phenomenex, 250 × 0.64 mm) using H_2_O-MeCN (1:1) giving compound **1** (1.7 mg), **3** (3.6 mg) and **6** (2.4 mg). Similarly, the cytotoxic fraction (F5, 17 mg) (IC_50_ = 3.5 µg/mL against HCT 116) was purified on C18 HPLC column (Gemini^®^ 5 µm, Phenomenex, 250 × 0.64 mm) using H_2_O-MeCN (6:4) to give **2** (2.7 mg), **4** (2.3 mg) and **5** (1.9 mg).

#### Spectroscopic Data of the Compounds

##### Pseudoceratonic acid (**1**)

Colorless oil; positive HRESIMS *m/z* 366.9064 (calcd for C_6_H_14_^79^Br_2_N_2_O_4_P, 366.9057, [M + H]^+^); negative HRESIMS *m/z* 364.8897 (calcd. for C_6_H_12_^79^Br_2_N_2_O_4_P, 364.8901, [M − H]^−^); NMR: Table 1.

##### Ceratinine N (**2**)

Colorless solid; UV (MeOH) λ_max_ (log ε): 2.15 (3.74), 227 (3.81), 276 (2.75), 284 (2.70) nm; HRESIMS *m/z* 509.9669 (calcd. for C_19_H_18_^79^Br_2_N_3_O_4_, [M + H]^+^, 509.9664); NMR: Table 2. 

##### Ceratinine O (**3**)

Colorless solid; UV (MeOH) λ_max_ (log ε): 2.16 (3.69), 230 (3.91), 276 (2.99), 284 (2.96) nm; HRESIMS *m/z* 422.9923 (calcd. for C_14_H_21_^79^Br_2_N_2_O_3_, [M + H]^+^, 422.9918), NMR: Table 2.

### 3.4. Biological Evaluation of the Compounds

#### Antimicrobial Evaluation of the Compounds

##### Disc Diffusion Assay

Using *S. aureus* (ATCC 25923), *E. coli* (ATCC 25922), and *C. albicans* (ATCC 14053), a disc diffusion assay was carried out to evaluate the antimicrobial effects of **1**–**6** at 50 µg/disc as reported before [35,36,37]. Ketoconazole (50 µg/disc) and ciprofloxacin (5.0 µg/disc) served as antibiotics controls.

##### Determination of the MIC of the Compounds

The MIC values of the compounds were determined of using a macrodilution method [38]. Briefly, the compounds were dissolved in MeOH at a final concentration of 2000 µg/mL. Ciprofloxacin and ketoconazole were dissolved in H_2_O at final concentrations of 100 µg/mL. Syringe filters (0.2 µm) were used for sterilization of all stock solutions. Two-fold serial dilutions of the solutions were used in Mueller Hinton Broth (MHB) to give concentrations of 1.0–1000 µg/mL (for the compounds) and 0.125–64 µg/mL (for ciprofloxacin and ketoconazole). 500 µL from the 10^6^ CFU/mL microbial suspensions were added in sterile tubes to give inoculua of 5 × 10^5^ CFU/mL. Further, 100 µL of the stock solutions of the compounds and antibiotics were added into the tubes. Control tubes with test microorganisms only and MeOH were prepared. The MeOH did not exhibit any antimicrobial activity. The tubes were kept for 48 h at 37 °C. MIC values were calculated as the lowest concentrations of the compounds/antibiotics, which did not exhibit any microbial growth. 

### 3.5. Determination of the Cytotoxicity of the Compounds

#### 3.5.1. Cell Lines and Cultures

The cell lines MDA-MB-231 (triple-negative breast cancer, ATCC HTB-26), HeLa (human cervical carcinoma, ATCC CCL-2), HCT 116 (colorectal carcinoma, ATCC CCL-247) were used for the evaluation of the cytotoxicity of the compounds. In addition, NHDF (normal human dermal fibroblasts) cells were used to evaluate the selectivity of compound **2** against the cancerous cell lines. DMEM, with Pen–Strep. (1%) and FBS (10%), was used for culturing MDA-MB-231 cells, while RPMI 1640, with Pen–Strep. (1%) and FBS (10%), was used for culturing HCT 116 and HeLa cells. Finally, NHDF cells were cultured in fibroblast basal medium complemented with Pen–Strep. (1% *v*/*v*), heat-inactivated fetal bovine serum (FBS) (2% *v*/*v*), insulin (5 µg/mL) and basic fibroblast growth factor (1 ng/mL). Culture of all cells was completed at 37 °C with 5% CO_2_ and 95% humidity [15,39].

#### 3.5.2. MTT Assay

The cytotoxic effects of the compounds were evaluated using MTT assay as reported earlier [15,39]. Briefly, all cells kept overnight in 5% CO_2_/air and at 37 °C. Tested compounds were added to the upper row of a 96-well plate followed by 1:4 (*v*/*v*) serial dilutions. After 72 h incubation of the compounds with the cells (10,000 cells/well for HCT 116 and NHDF, and 12,000 cells/well for HeLa and MDA-MB-231), the cells’ viability was calculated at 490 nm as reported before [15,39]. 5-Fluorouracil (5-FU) and DMSO were used as positive and negative controls. The IC_50_ values reported in Table 3 represent the mean of three separate experiments. A concentration of 10 µM was set as a cutoff value in this assay.

## 4. Conclusions

Purification of the cytotoxic and antibacterial fractions of the extract of the sponge *Pseudoceratina arabica* gave six compounds including pseudoceratonic acid (**1**), ceratinines N (**2**) and O (**3**) along with molka’iamine (**4**), hydroxymolka’iamine (**5**) and ceratinamine (**6**). Further, the structures of **1**–**6** were assigned by NMR and HRESIMS spectral interpretations. Pseudoceratonic acid possesses an unprecedented *N,N*-dibromo-phosphorohydrazidic acid that was not encountered previously. The existence of pseudoceratonic acid provides insight into unprecedented biochemical transformations within the sponge *P. arabica*. The stability of this previously unknown functional group also suggests a new functional group that might be used as an isostere for application in synthetic and medicinal chemistry or as a linking group for other design purposes. Pseudoceratonic acid displayed selective antibiotic activity against *S. aureus* and *E. coli*, while ceratinine N was selective against *C. albicans*. Further, creatinine N was potently active, with a marginal selectivity, against three cancerous human cell lines down to 2.3 µM. This potent effect is attributed to the existence of 2-(methylene)cyclopent-4-ene-1,3-dione moiety in the compound. Thus, creatinine N represents a potential scaffold for the development of novel and more effective anticancer drug leads.

## Figures and Tables

**Table 1:** NMR data of pseudoceratonic acid (**1**) (CD_3_OD) ^a^.

No.	δ_C_ (mult.)	δ_N_^b^	δ_H_ (mult.)	^1^H-^13^C HMBC	^1^H-^15^N HMBC
1	31.0, CH_3_		2.23 (3H, s)	C-2, C-3	
2	209.3, qC				
3	50.7, CH_2_		2.91 (2H, s)	C-2, C-4, C-6	*N*-7
4	53.4, qC				
5	26.0, CH_3_		1.42 (3H, s)	C-3, C-4	*N*-7
6	26.0, CH_3_		1.42 (3H, s)	C-3, C-4	*N*-7
*N*-7		−331.6			

^a 1^H and ^13^C NMR were acquired at 400 and 100 MHz, respectively. ^b 15^N Signal was traced from ^1^H-^15^N HMBC and was referenced to CH_3_NO_2_ at 0.0 ppm.

**Table 2:** NMR data of ceratinines N (**2**) and O (**3**) (CD_3_OD) ^a^.

No.	2			3		
	δ_C_ (Mult.) ^a^	δ_H_ (Mult., *J* (Hz))	HMBC (C#)	δ_C_ (Mult.) ^a^	δ_H_ (Mult., *J* (Hz))	HMBC (C#)
1	138.6 (qC)			139.6 (qC)		
2	134.8 (CH)	7.45 (s)	C-1, C-3/5, C-4, C-7	134.3 (CH)	7.46 (s)	C-1, C-3/5, C-4, C-7
3	119.3 (qC)			119.1 (qC)		
4	153.2 (qC)			152.9 (qC)		
5	119.3 (qC)			119.1 (qC)		
6	134.8 (CH)	7.45 (s)	C-1, C-3/5, C-4, C-7	134.3 (CH)	7.46 (s)	C-1, C-3/5, C-4, C-7
7	36.7 (CH_2_)	2.86 (t, 6.6)	C-1, C-2/6, C-8	33.0 (CH_2_)	2.92 (t, 6.6)	C-1, C-2/6, C-8
8	51.9 (CH_2_)	3.61 (t, 6.6)	C-1, C-7, C-12	41.0 (CH_2_)	3.13 (t, 6.6)	C-1, C-7
9	71.0 (CH_2_)	4.02 (t, 6.6)	C-4, C-10, C-11	72.0 (CH_2_)	4.01 (t, 6.6)	C-4, C-10, C-11
10	30.1 (CH_2_)	2.08 (quin, 6.6)	C-9, C-11	31.3 (CH_2_)	2.01 (quin, 6.6)	C-9, C-11
11	38.4 (CH_2_)	3.56 (t, 6.6)	C-9, C-10, C-18	39.1 (CH_2_)	3.42 (t, 6.6)	C-9, C-10, C-12
12	150.8 (CH)	7.21 (s)	C-8, C-13, C-14, C-17	159.2 (qC)		
13	99.5 (qC)			61.7 (CH_2_)	4.05 (q, 6.6)	C-12, C-14
14	198.7 (qC)			14.5 (CH_3_)	1.22 (t, 6.6)	C-13
15	142.7 (CH)	6.67 (d, 6.6)	C-13, C-14, C-16, C-17			
16	142.9 (CH)	6.73 (d, 6.6)	C-13, C-14, C-15, C-17			
17	196.8 (qC)					
18	145.0 (qC)					
19	113.1 (qC)					

^a^ Data acquired at 600 and 150 MHz for ^1^H and ^13^C, respectively; # means No.; C# = (Carbon No.).

**Table 3:** Antimicrobial effects of **1**–**6**.

Compound	*E. coli*		*S. aureus*		*C. albicans*	
	Inhibition Zone (mm)	MIC (µg/mL)	Inhibition Zone (mm)	MIC (µg/mL)	Inhibition Zone (mm)	MIC (µg/mL)
**1**	15	16	17	16	6	125
**2**	6	125	7	125	16	16
**3**	6	125	8	64	9	64
**4**	6	125	7	125	6	125
**5**	NT	NT	NT	NT	NT	NT
**6**	7	125	9	64	12	32
Ciprofloxacin ^a^	30	0.25	22	0.5	NT	NT
Ketoconazole ^b^	NT	NT	NT	NT	30	0.5

^a^ positive antibacterial drug; ^b^ positive antifungal drug; NT = Not Tested.

**Table 4:** Cytotoxic effects of **1**–**6.** ^a^

Compound	IC_50_ (µM)			
	MDA-MB-231	HeLa	HCT 116	NHDF
**1**	≥10	≥10	≥10	
**2**	3.1 ± 0.29	2.3 ± 0.16	2.9 ± 0.18	3.5 ± 0.30
**3**	≥10	≥10	9.5 ± 0.85	
**4**	≥10	≥10	≥10	
**5**	≥10	≥10	≥10	
**6**	≥10	≥10	≥10	
5-FU ^b^	13.0 ± 0.30	12.3 ± 0.25	4.6 ± 0.23	

^a^ Cells were treated with the compounds for 72 h; ^b^ Positive cytotoxic drug; NHDF: normal human dermal fibroblasts.

## Supplementary Materials

The following are available online at https://www.mdpi.com/1660-3397/18/11/525/s1, ^1^H NMR, ^13^C NMR, DEPT, COSY, HSQC, ^1^H-^13^C HMBC, and HRESIMS data of compounds **1**–**3** and ^1^H-^15^N HMBC of compound **1**.
