# Supplementary material for: Pseudoceratonic Acid and Moloka’iamine Derivatives from the Red Sea Verongiid Sponge Pseudoceratina arabica
Source: Mar Drugs. 2020 Oct 23;18(11):525. doi: 10.3390/md18110525 (PMC7690883; doi:10.3390/md18110525)
Supplement: Supplementary file 1 [file marinedrugs-18-00525-s001.pdf]

Supplementary Information

Figure S1. Positive HRESIMS of pseudoceratonic acid (**1**) S1
Figure S2. Negative HRESIMS of pseudoceratonic acid (**1**) S1
Figure S3. ^{1}H NMR spectrum of pseudoceratonic acid (**1**) (CD_3OD) S2
Figure S4. ^{13}C NMR spectrum of pseudoceratonic acid (**1**) (CD_3OD) S2
Figure S5. HSQC spectrum of pseudoceratonic acid (**1**) (CD_3OD) S3
Figure S6. ^{1}H-^{13}C HMBC spectrum of pseudoceratonic acid (**1**) (CD_3OD) S3
Figure S7. ^{1}H-^{15}N HMBC spectrum of pseudoceratonic acid (**1**) (CD_3OD) S4
Figure S8. LRESIMS and fragmentation ion peaks of pseudoceratonic acid (**1**) S4
Figure S9. Positive HRESIMS of ceratinine N (**2**) S5
Figure S10. ^{1}HNMR spectrum of ceratinine N (**2**) (CD_3OD) S5
Figure S11. ^{13}C NMR spectrum of ceratinine N (**2**) (CD_3OD) S6
Figure S12. DEPT spectrum of ceratinine N (**2**) (CD_3OD) S6
Figure S13. COSY spectrum of ceratinine N (**2**) (CD_3OD) S7
Figure S14. HSQC spectrum of ceratinine N (**2**) (CD_3OD) S7
Figure S15. HMBC spectrum of ceratinine N (**2**) (CD_3OD) S8
Figure S16. Positive HRESIMS of ceratinine O (**3**) S8
Figure S17. ^{1}H NMR spectrum of ceratinine O (**3**) (CD_3OD) S9
Figure S18. ^{13}C NMR spectrum of ceratinine O (**3**) (CD_3OD) S9
Figure S19. DEPT spectrum of ceratinine O (**3**) (CD_3OD) S10
Figure S20. COSY spectrum of ceratinine O (**3**) (CD_3OD). S10
Figure S21. HSQC spectrum of ceratinine O (**3**) (CD_3OD) S11
Figure S22. HMBC spectrum of ceratinine O (**3**) (CD_3OD) S11

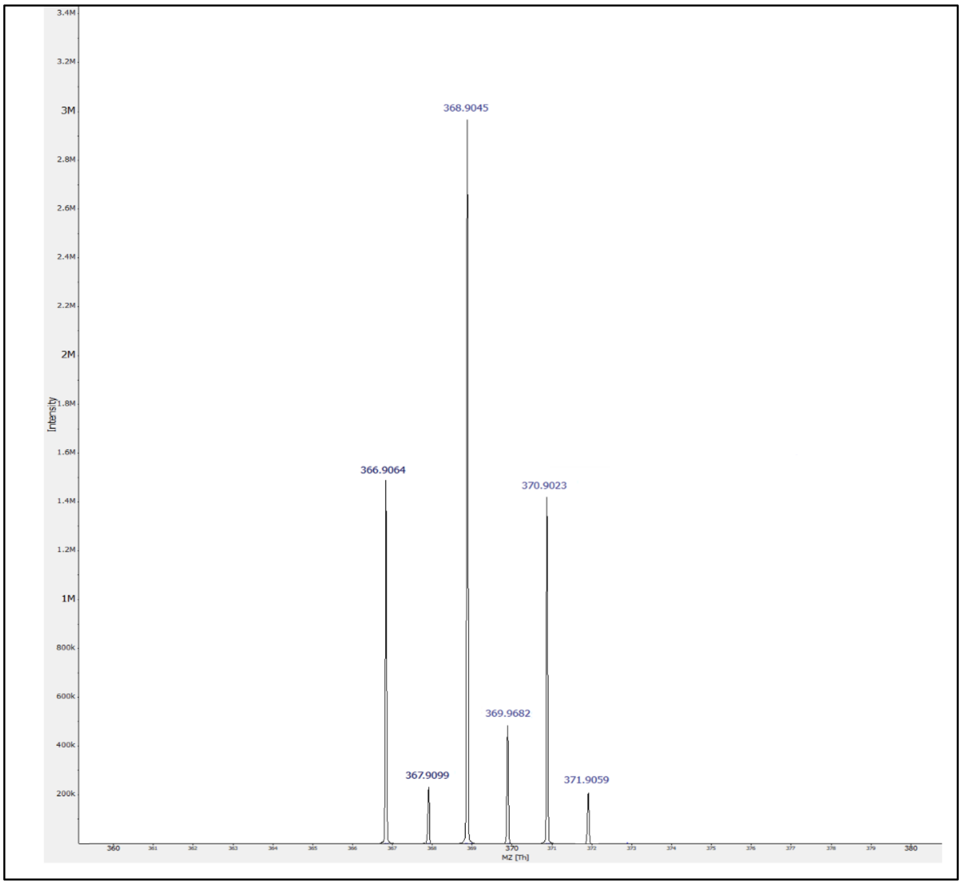

Figure S1. Positive HRESIMS of pseudoceratonic acid (**1**).

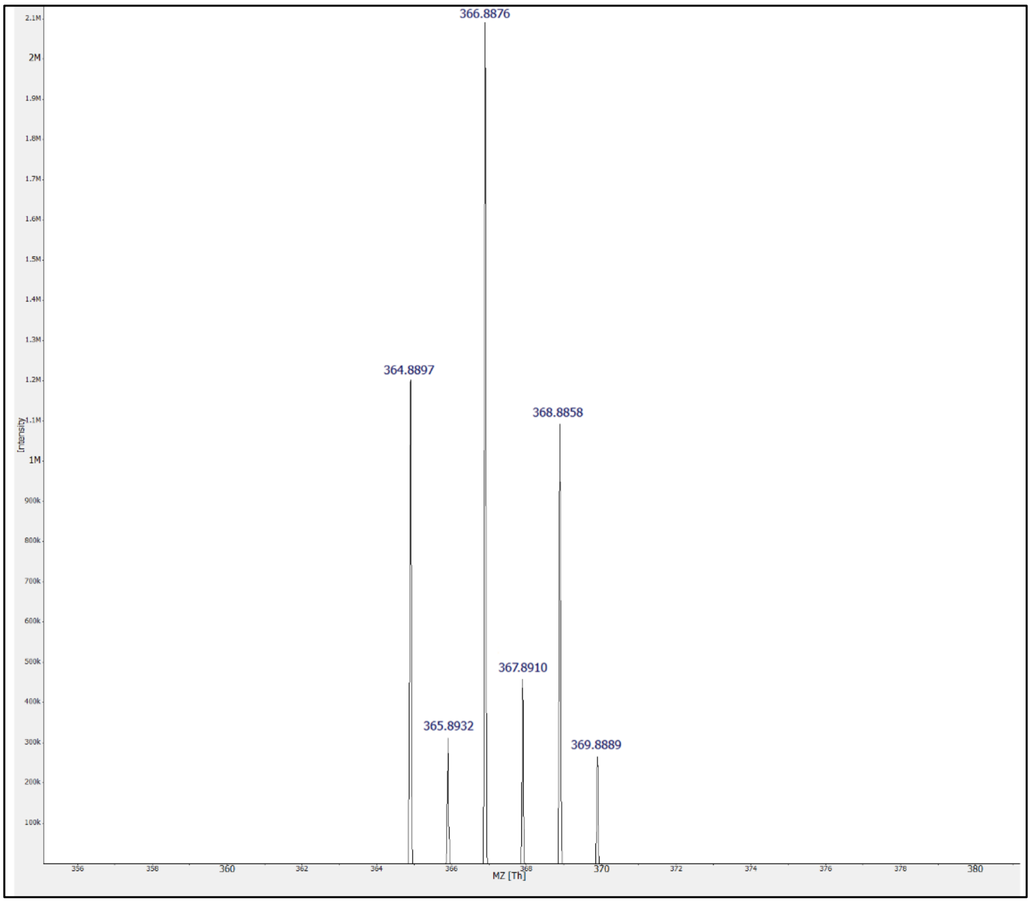

Figure S2. Negative HRESIMS of pseudoceratonic acid (**1**).

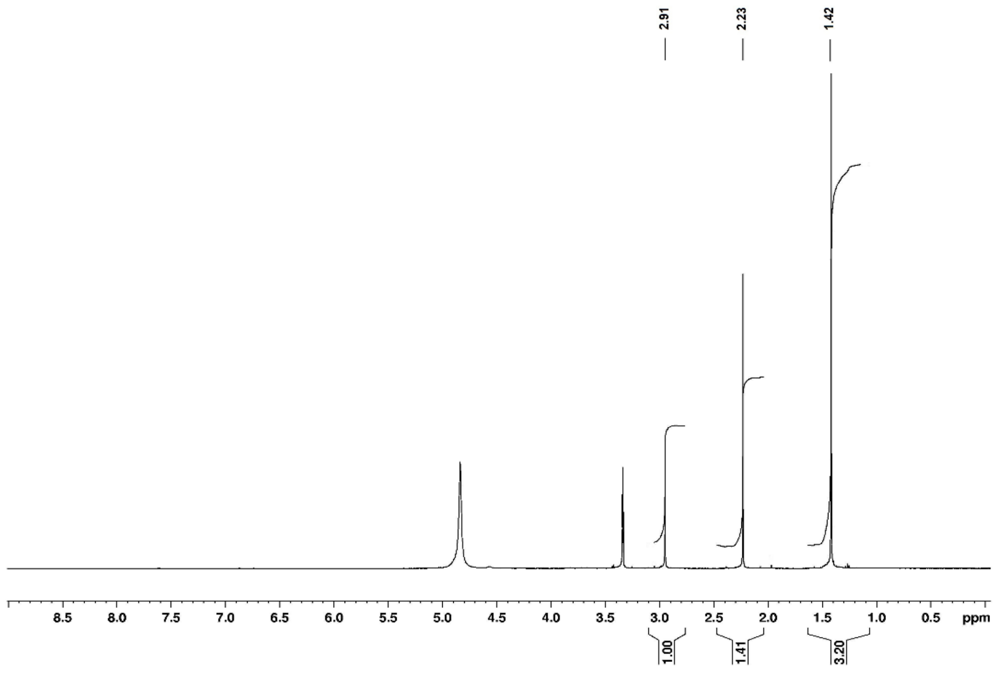

Figure S3. ^{1}H NMR spectrum of pseudoceratonic acid (**1**) (CD_3OD).

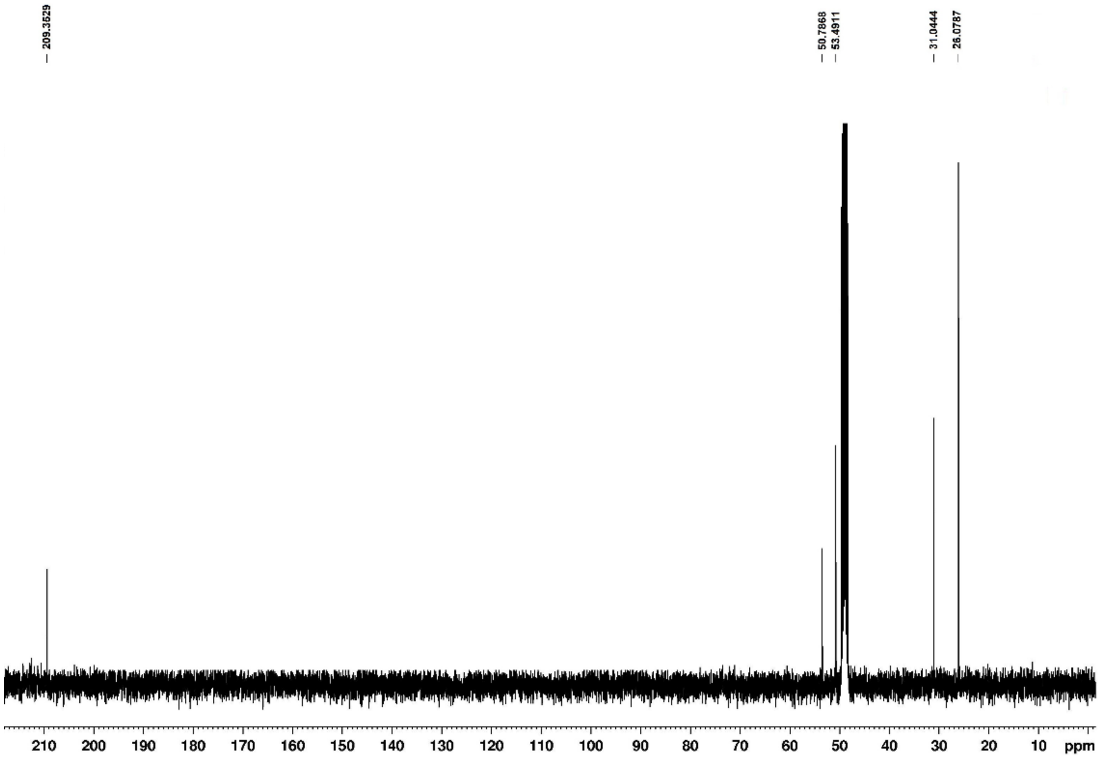

Figure S4. ^{13}C NMR spectrum of pseudoceratonic acid (**1**) (CD_3OD).

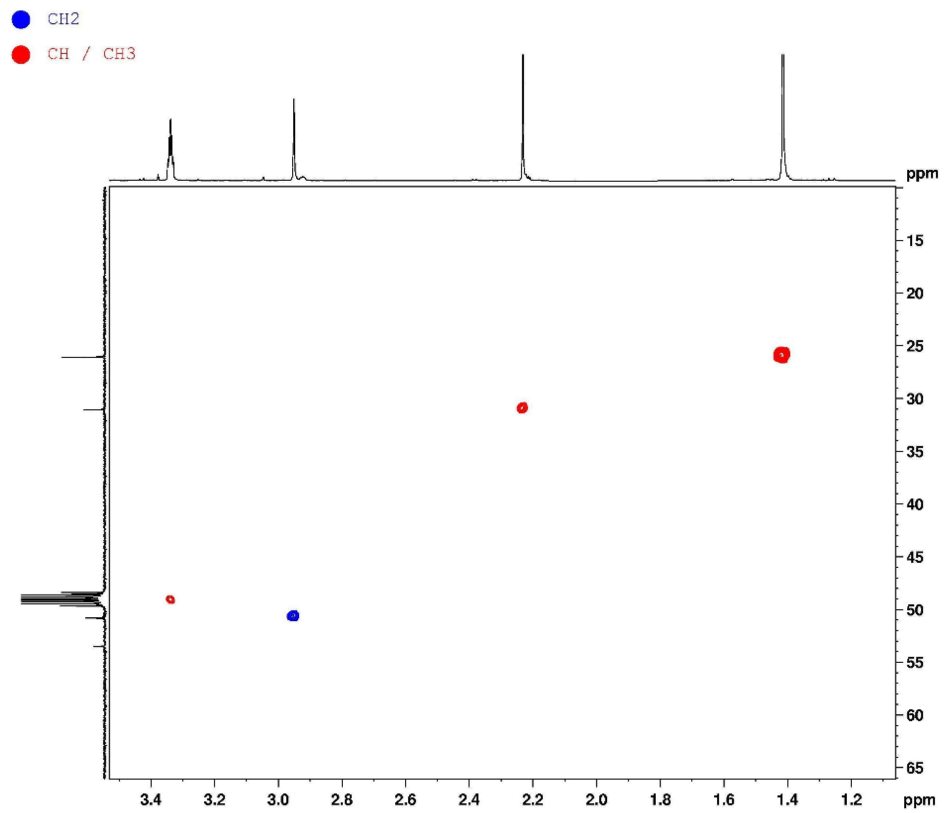

Figure S5. HSQC spectrum of pseudoceratonic acid (**1**) (CD_3OD).

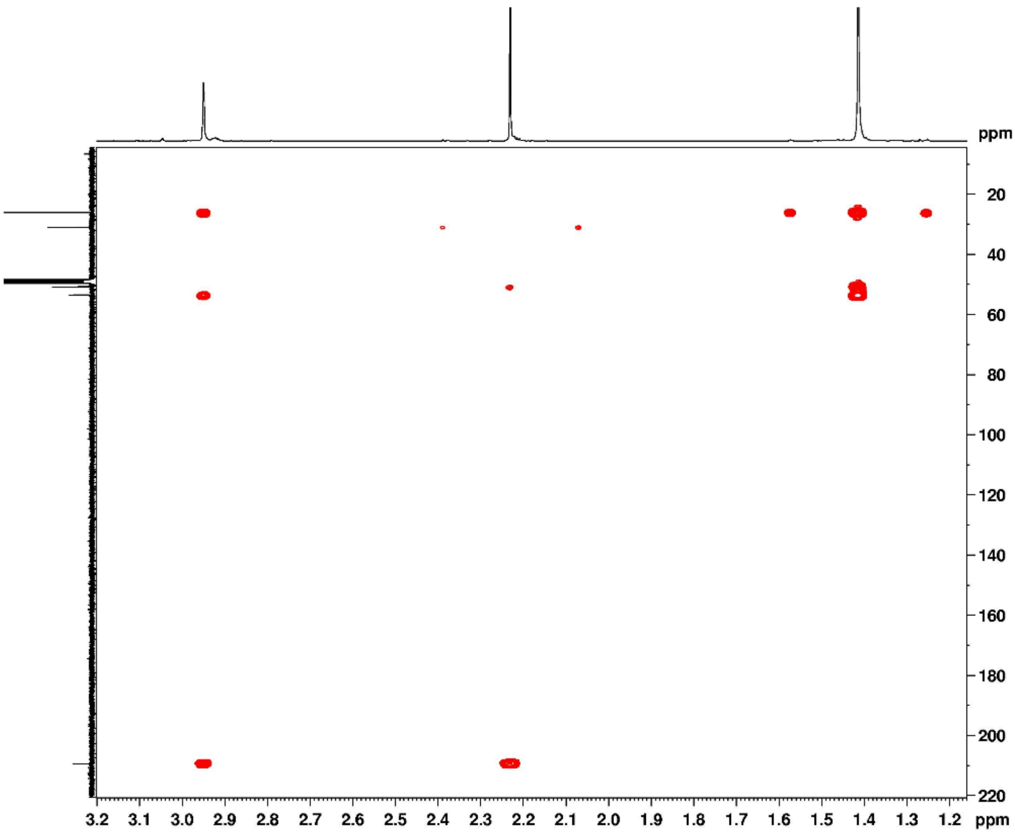

Figure S6. ^{1}H-^{13}C HMBC spectrum of pseudoceratonic acid (**1**) (CD_3OD).

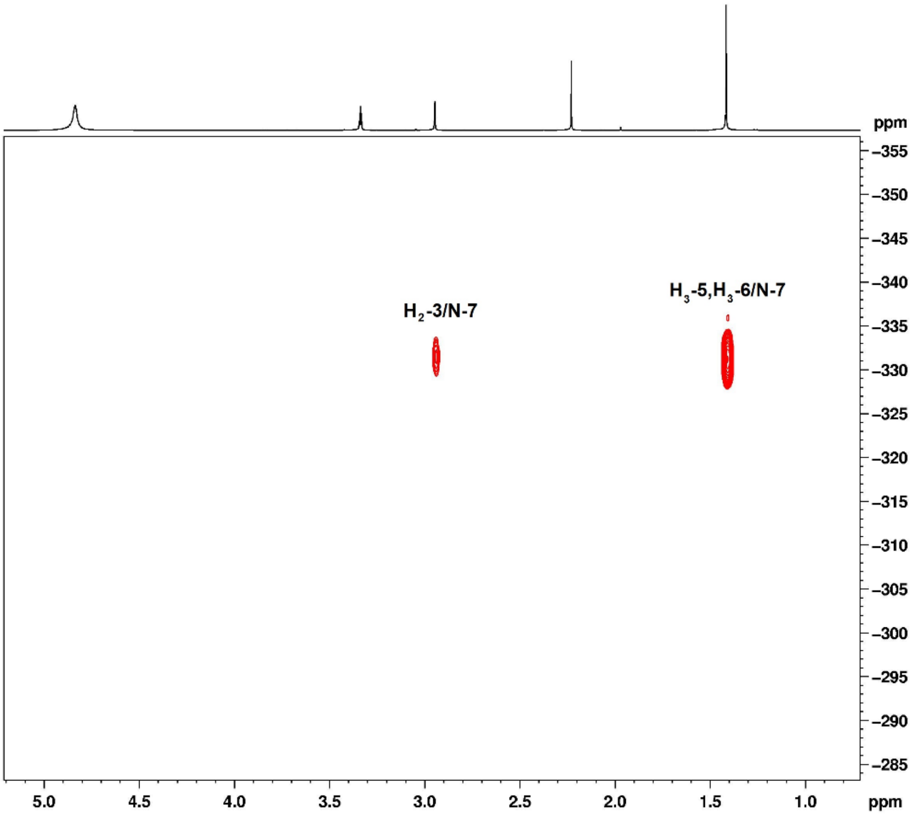

Figure S7. ^{1}H-^{15}N HMBC spectrum of pseudoceratonic acid (**1**) (CD_3OD).

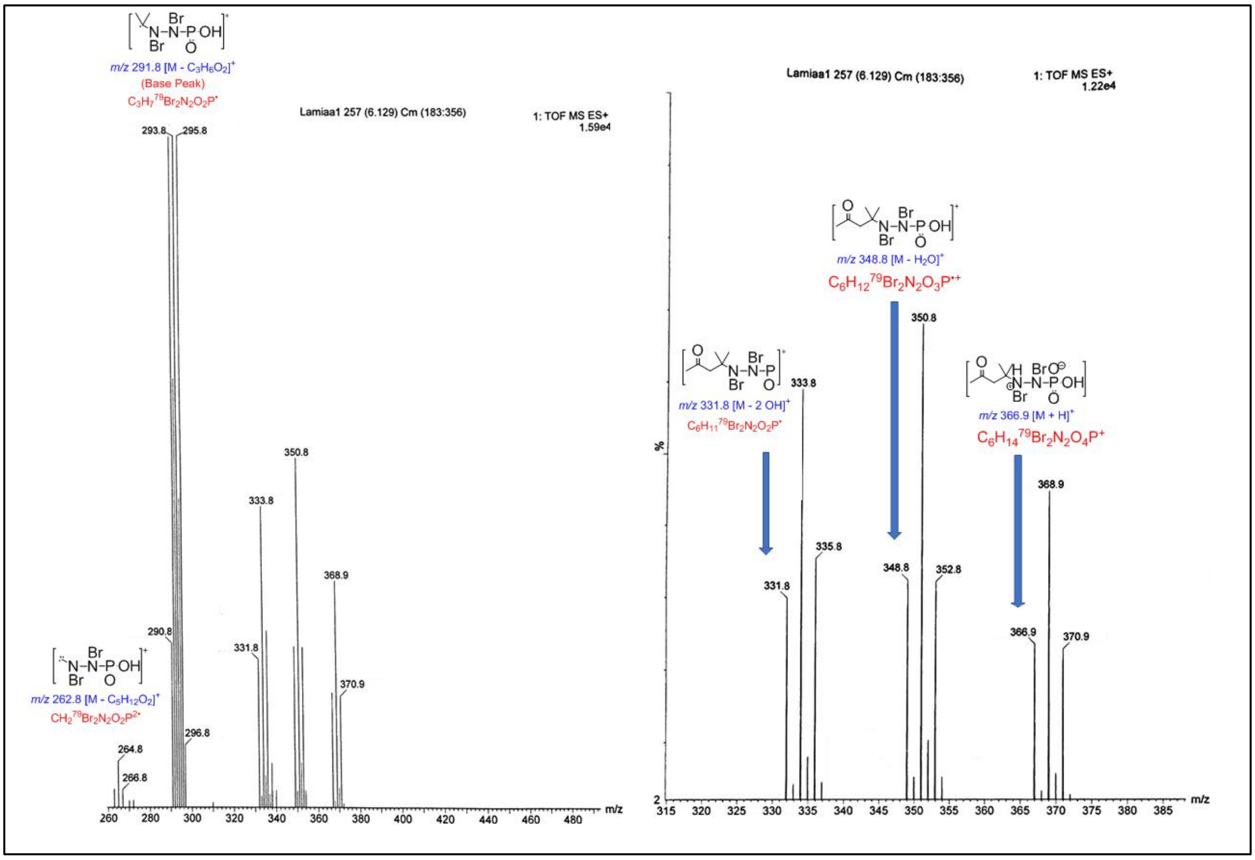

Figure S8. LRESIMS and fragmentation ion peaks of pseudoceratonic acid (**1**).

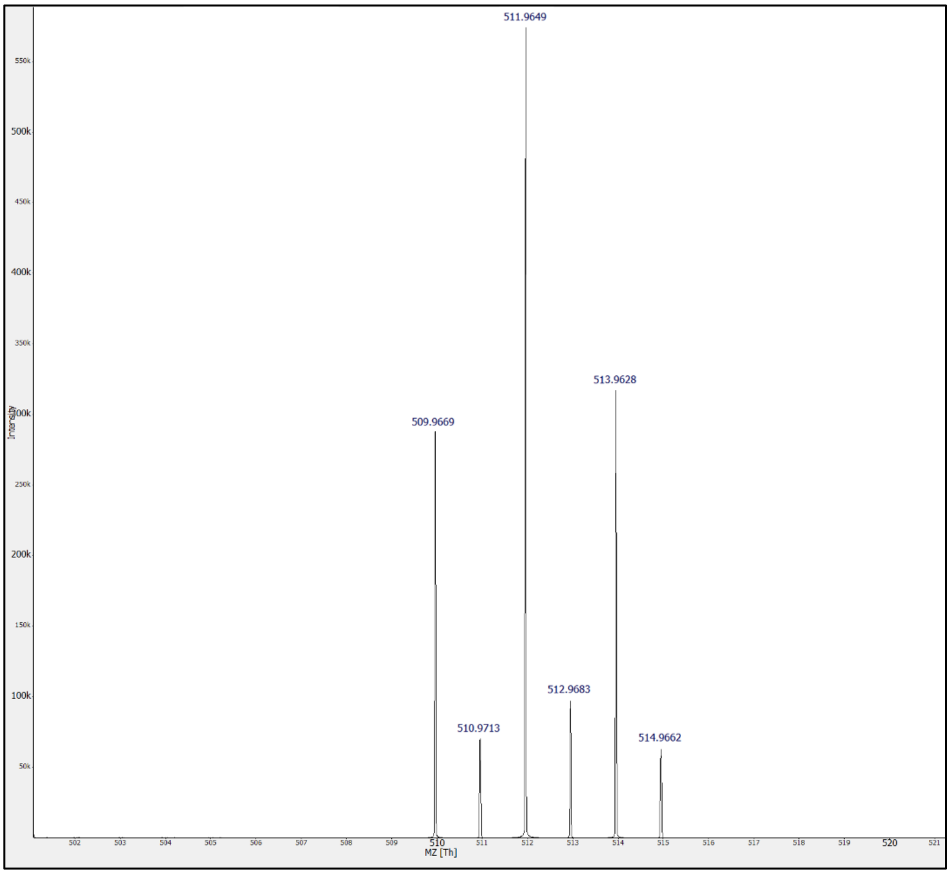

Figure S9. Positive HRESIMS of ceratinine N (**2**).

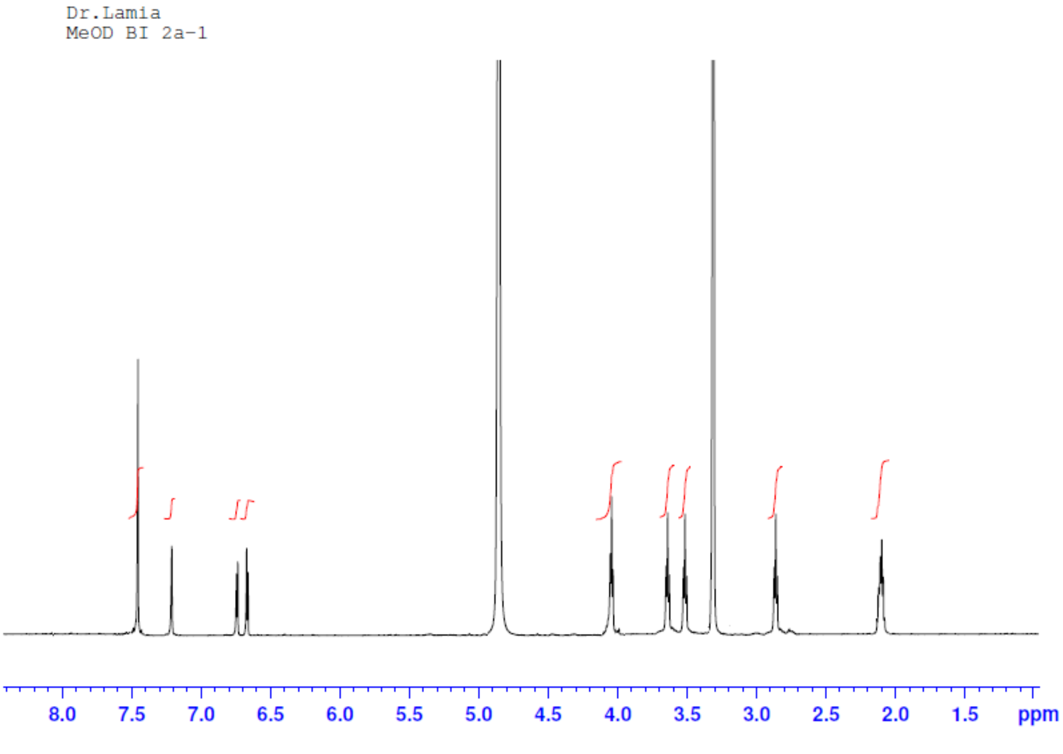

Figure S10. ^{1}H NMR spectrum of ceratinine N (**2**) (CD_3OD).

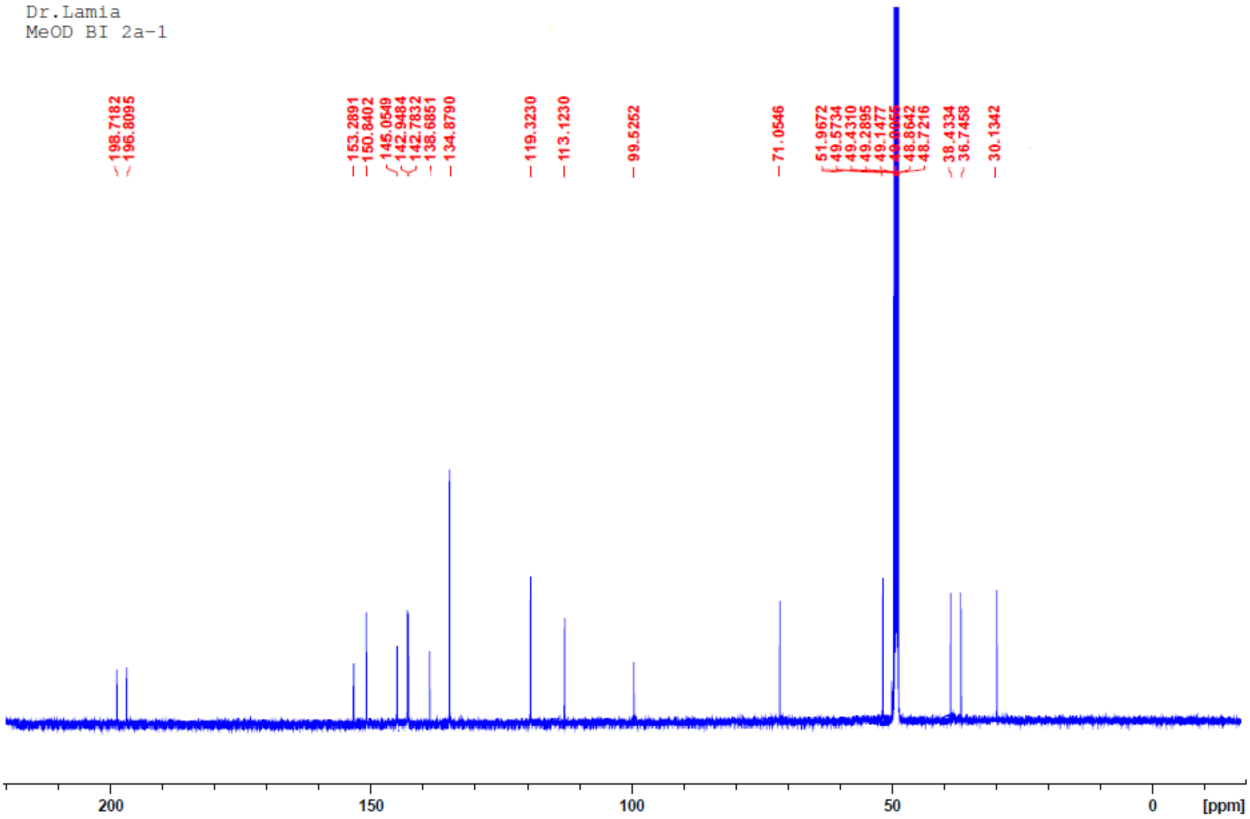

Figure S11. [13]H NMR spectrum of ceratinine N (**2**) (CD_3OD).

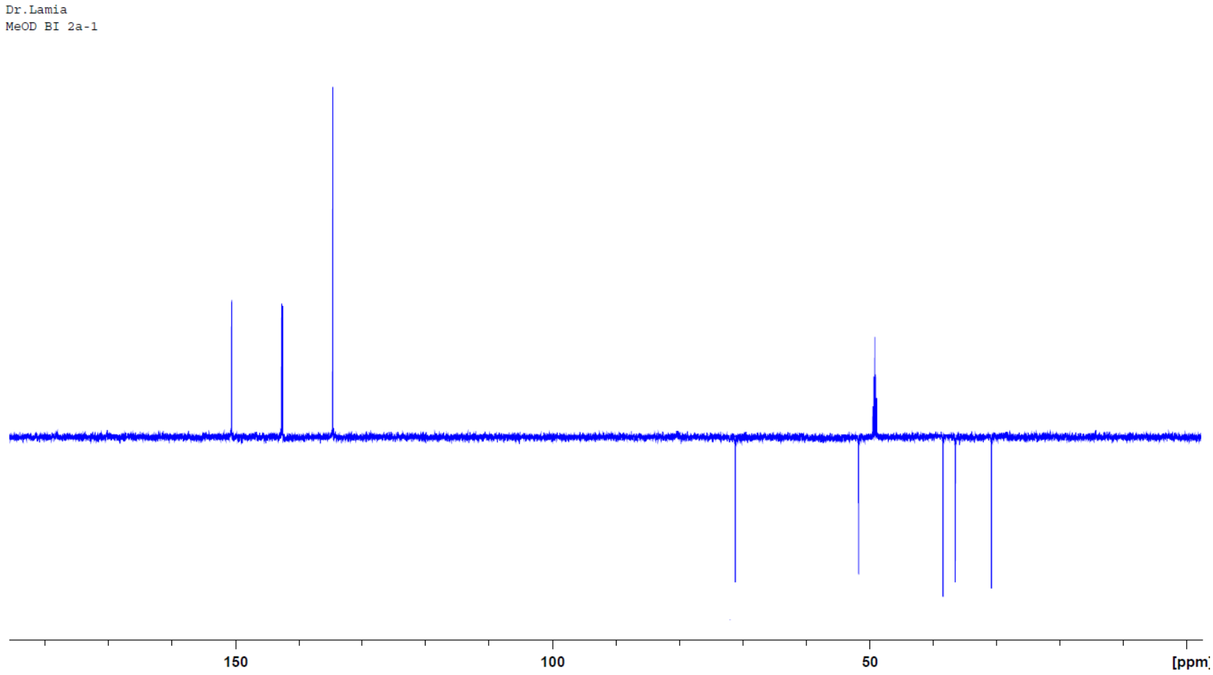

Figure S12. DEPT spectrum of ceratinine N (**2**) (CD_3OD).

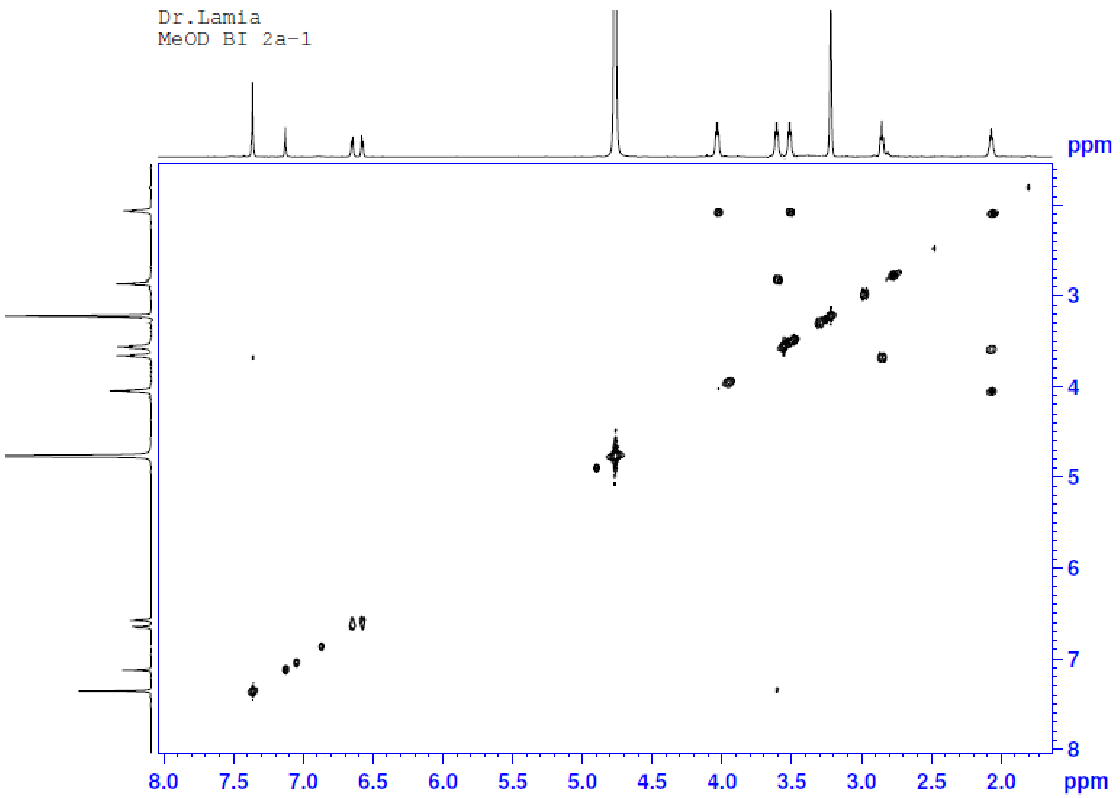

Figure S13. COSY spectrum of ceratinine N (**2**) (CD_3OD).

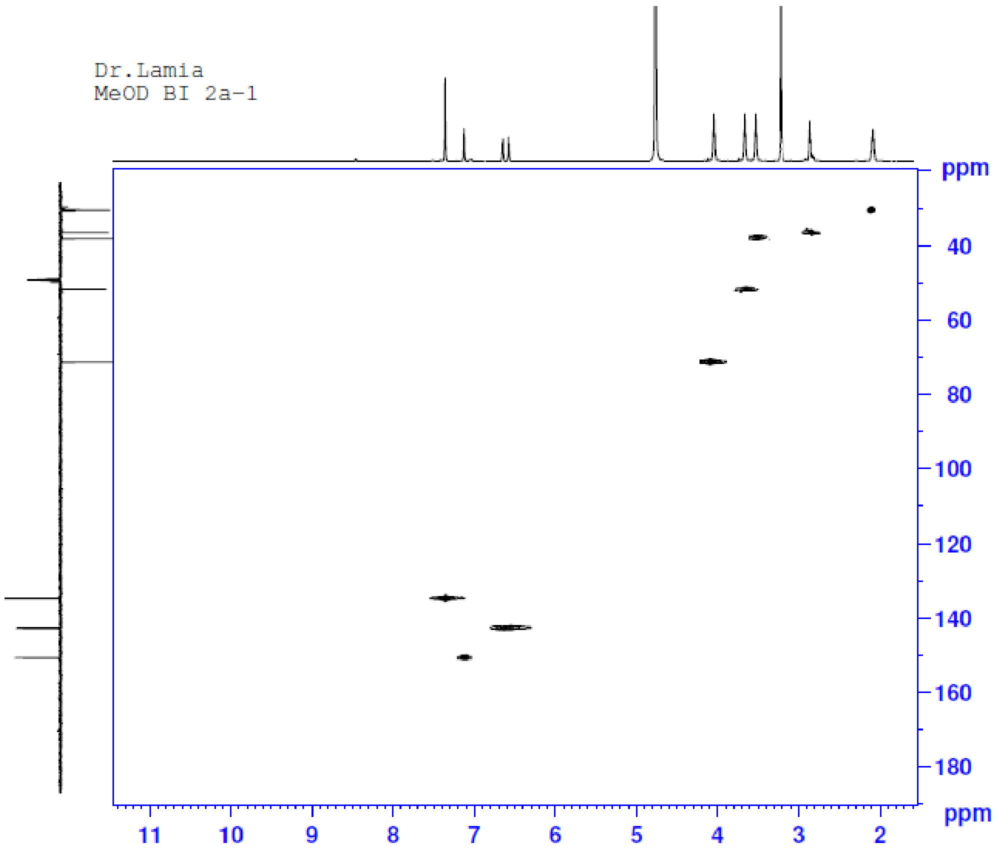

Figure S14. HSQC spectrum of ceratinine N (**2**) (CD_3OD).

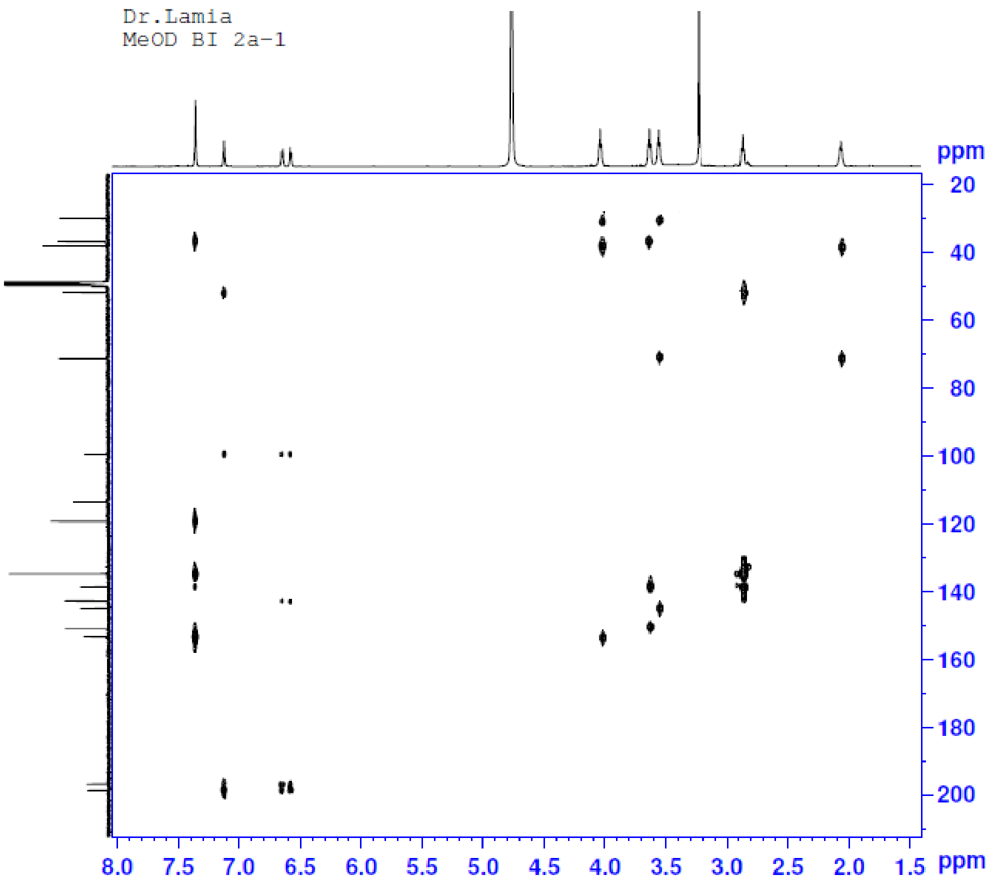

Figure S15. HMBC spectrum of ceratinine N (**2**) (CD_3OD).

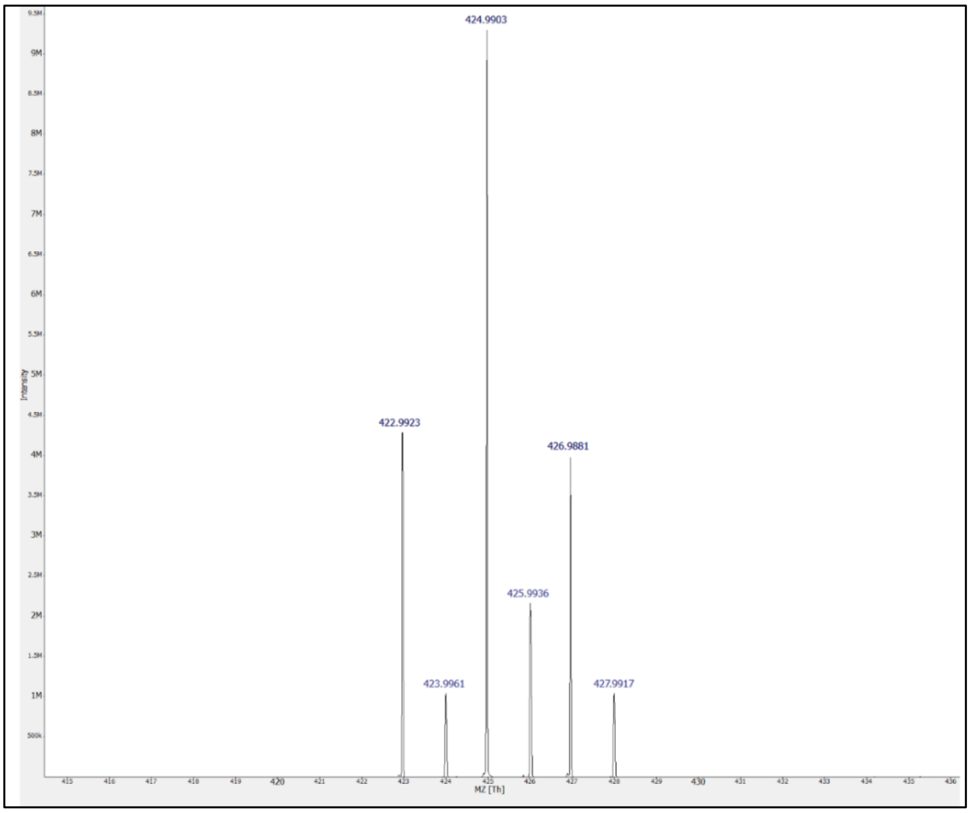

Figure S16. Positive HRESIMS of ceratinine O (**3**).

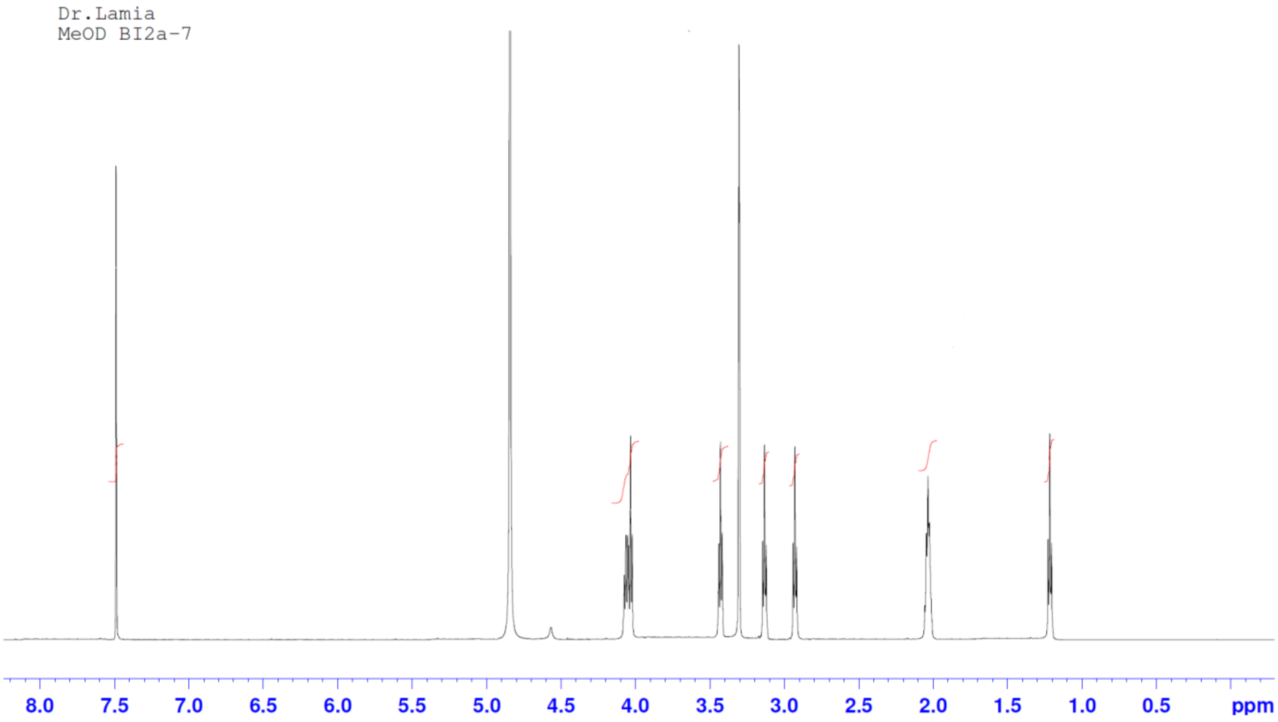

Figure S17. ^{1}H NMR spectrum of ceratinine O (**3**) (CD_3OD).

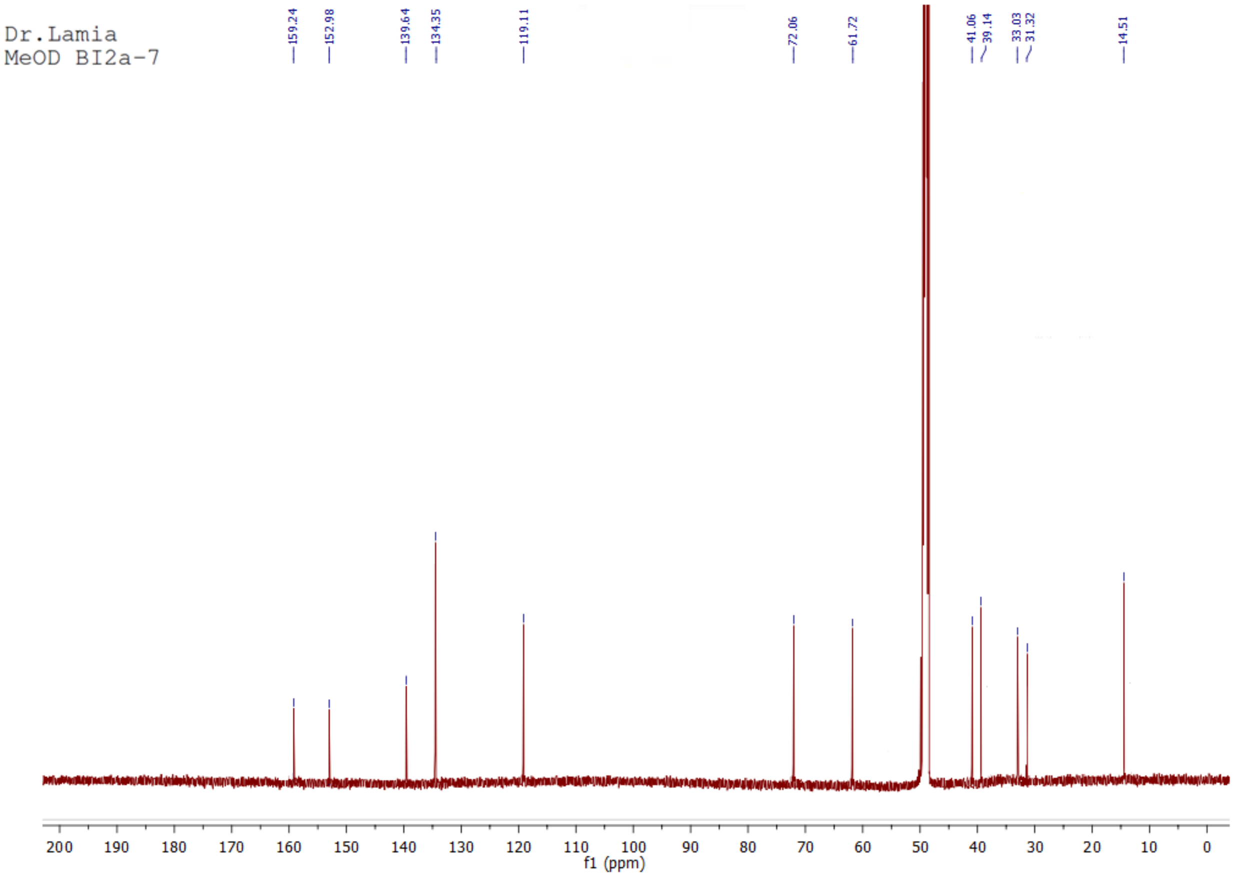

Figure S18. ^{13}C NMR spectrum of ceratinine O (**3**) (CD_3OD).

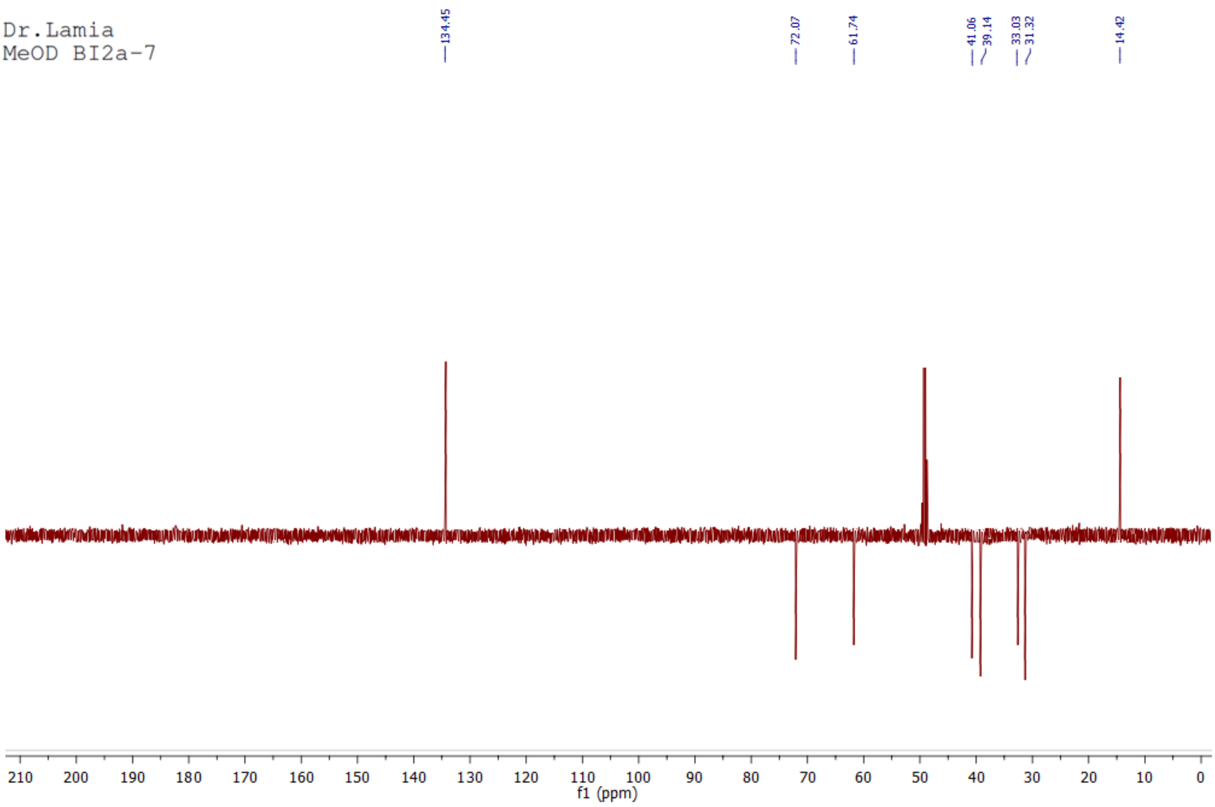

Figure S19. DEPT spectrum of ceratinine O (**3**) (CD_3OD).

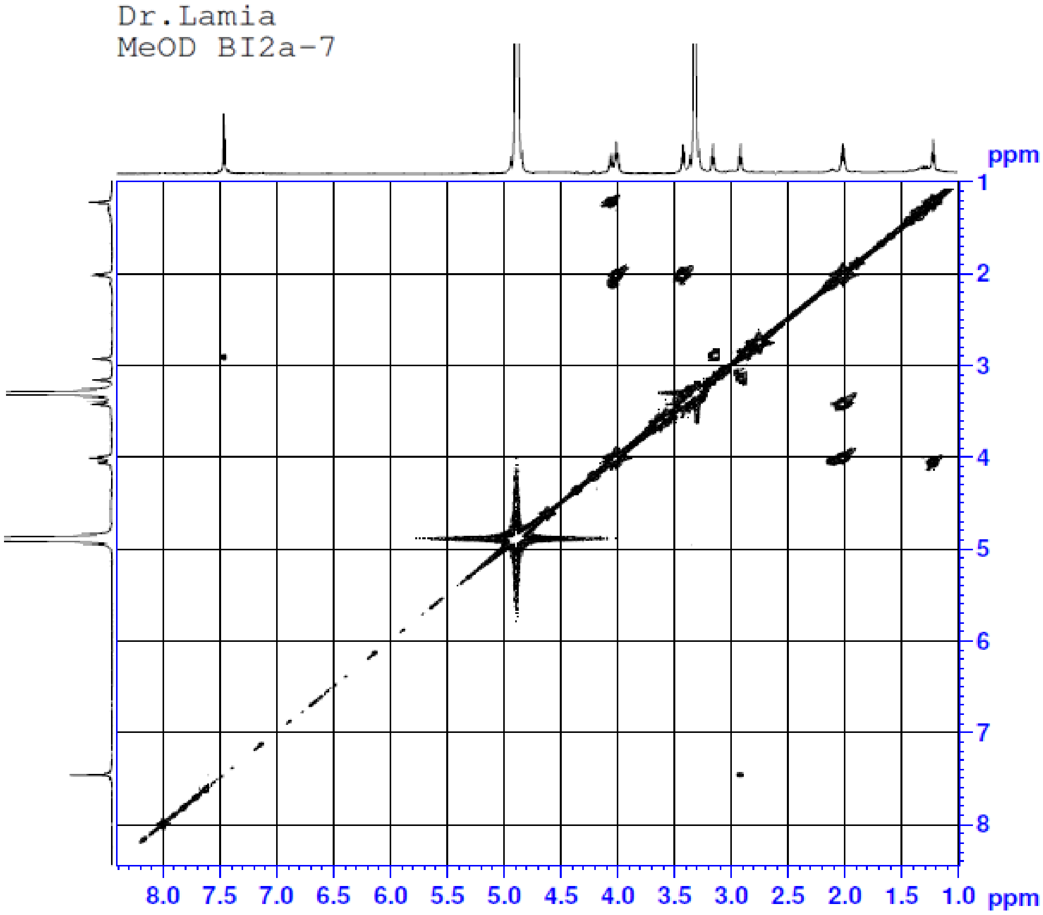

Figure S20. COSY spectrum of ceratinine O (**3**) (CD_3OD).

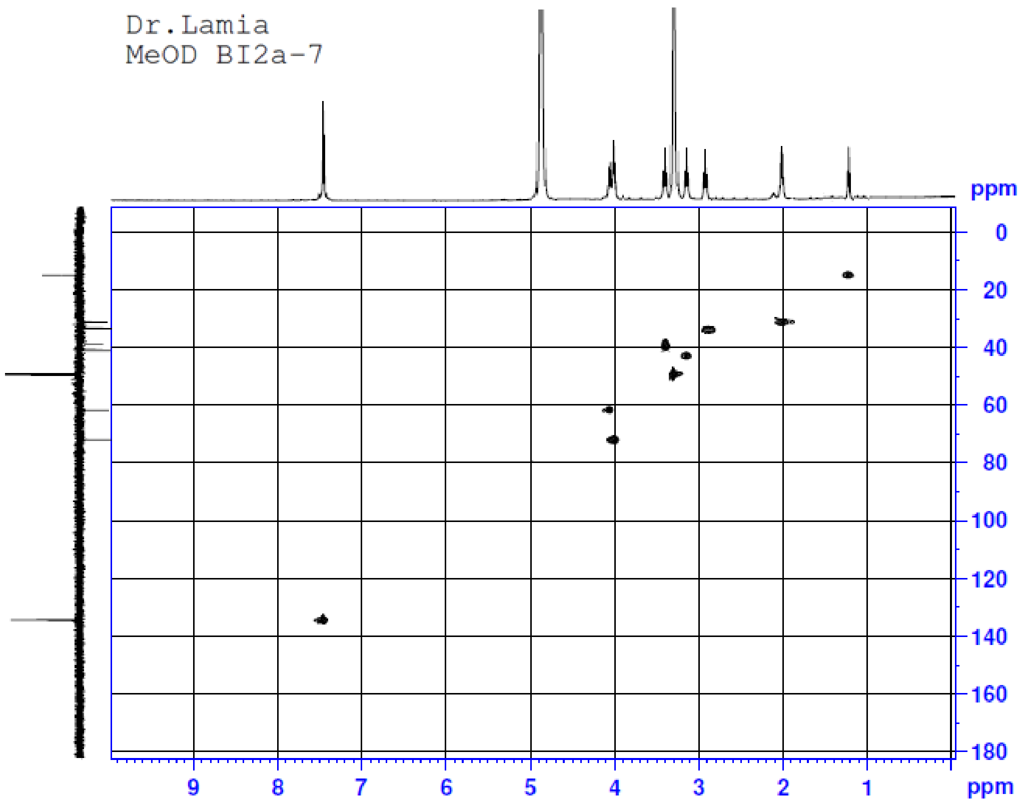

Figure S21. HSQC spectrum of ceratinine O (**3**) (CD_3OD).

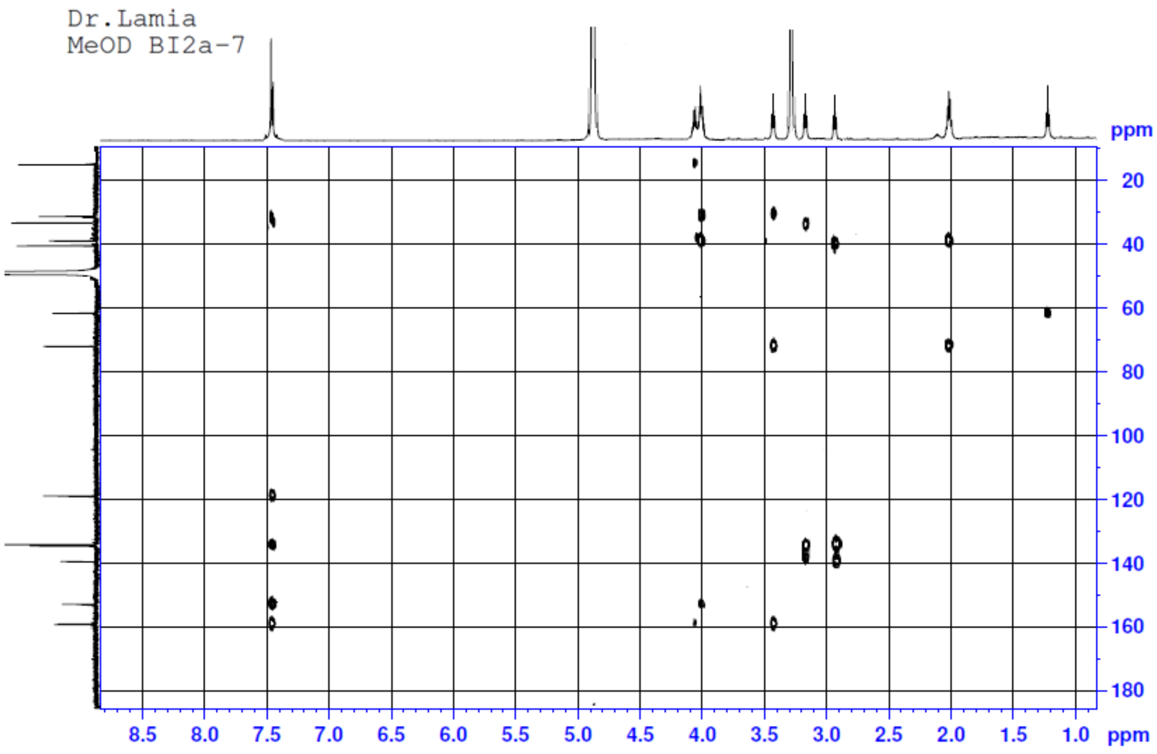

Figure S22. HMBC spectrum of ceratinine O (**3**) (CD_3OD).
